# Radiation-induced Bystander Effect (RIBE) alters mitochondrial metabolism using a human rectal cancer *ex vivo* explant model

**DOI:** 10.1016/j.tranon.2020.100882

**Published:** 2020-10-23

**Authors:** Aisling B. Heeran, Helen P. Berrigan, Croí E. Buckley, Heleena Moni Bottu, Orla Prendiville, Amy M. Buckley, Niamh Clarke, Noel E. Donlon, Timothy S. Nugent, Michael Durand, Cara Dunne, John O. Larkin, Brian Mehigan, Paul McCormick, Lorraine Brennan, Niamh Lynam-Lennon, Jacintha O'Sullivan

**Affiliations:** a Department of Surgery, Trinity Translational Medicine Institute, St. James's Hospital, Trinity College Dublin, Dublin 8, Ireland; b Institute of Food and Health and Conway Institute, UCD School of Agriculture and Food Science, UCD, Belfield, Dublin 4, Ireland; c GEMS, St. James's Hospital, Dublin 8, Ireland

**Keywords:** ATP, Adenosine triphosphate, CPMG, Carr-Purcell-Meiboom-Gill, CRC, colorectal cancer, CT, computed tomography, D_2_O, deuterium oxide, ECAR, extracellular acidification rate, Gy, Gray, ^1^H NMR, proton nuclear magnetic resonance, LARC, locally advanced rectal cancer, mtMP, mitochondrial membrane potential, NCM, normal conditioned media, neo-CRT, neoadjuvant chemoradiation, OAC, oesophageal adenocarcinoma, OCR, oxygen consumption rate, OPLS-DA, orthogonal partial least square discriminate analysis, OXPHOS, oxidative phosphorylation, PCA, principal component analysis, pCR, complete pathological response, PLS-DA, partial least square discriminate analysis, RIBE, Radiation-induced Bystander Effect, ROS, reactive oxygen species, TCM, tumour conditioned media, TME, Tumour microenvironment, TSP, sodium trimethyl [2,2,3,3-^2^H_4_] propionate, VFA, visceral fat area, Radiation-induced Bystander Effect, Rectal cancer, Metabolism, Radiation, Metabolomics

## Abstract

Locally advanced rectal cancer is treated with neoadjuvant-chemoradiotherapy, however only 22% of patients achieve a complete response. Resistance mechanisms are poorly understood. Radiation-induced Bystander Effect (RIBE) describes the effect of radiation on neighbouring unirradiated cells. We investigated the effects of *ex vivo* RIBE-induction from normal and rectal cancer tissue on bystander cell metabolism, mitochondrial function and metabolomic profiling. We correlated bystander events to patient clinical characteristics.

*Ex vivo* RIBE-induction caused metabolic alterations in bystander cells, specifically reductions in OXPHOS following RIBE-induction in normal (*p* = 0.01) and cancer tissue (*p* = 0.03) and reduced glycolysis following RIBE-induction in cancer tissue (*p* = 0.01). Visceral fat area correlated with glycolysis (*p* = 0.02) and ATP production (*p* = 0.03) following exposure of cells to TCM from irradiated cancer biopsies. Leucine levels were reduced in the irradiated cancer compared to the irradiated normal secretome (*p* = 0.04). ROS levels were higher in cells exposed to the cancer compared to the normal secretome (*p* = 0.04).

RIBE-induction *ex vivo* causes alterations in the metabolome in normal and malignant rectal tissue along with metabolic alterations in bystander cellular metabolism. This may offer greater understanding of the effects of RIBE on metabolism, mitochondrial function and the secreted metabolome.

## Highlights

• RIBE induction *ex vivo* alters mitochondrial metabolism in bystander cells.
• Rectal cancer secretome increases ROS in bystander cells.
• Higher leucine levels in the irradiated normal rectal secretome compared to the irradiated rectal cancer secretome
• Glycolysis and ATP levels in bystander cells correlate with patient's visceral fat area.

## Introduction

Colorectal cancer (CRC) is the 3rd most commonly diagnosed cancer globally and the 2nd leading cause of cancer death [1]. Within the European Union, rectal cancer accounts for approximately 35% of all cases of CRC [2]. The incidence of rectal cancer in Ireland is projected to almost double in both genders between 2020 and 2045 [3], while the mortality rate is predicted to increase by almost 25% by 2035 [4].

The current treatment for locally advanced rectal cancer (LARC) is neoadjuvant radiotherapy with or without chemotherapy, followed by surgery. Radioresistance is a considerable problem in rectal cancer with only 22% of patients achieving a complete pathological response (pCR) to neoadjuvant chemoradiation (neo-CRT) [5], which is the best predictor of long-term patient outcome [6]. The mechanisms underlying this resistance are poorly understood and the effect of radiation on bystander cells adjacent to the irradiated volume is largely unknown.

The Radiation-induced Bystander Effect (RIBE) refers to the plethora of biological phenomena occurring in non-irradiated cells as a result of signal transmission from an irradiated cell. Cell behaviour mimics that of directly irradiated cells, exhibiting genomic instability, mutagenesis, altered apoptosis, DNA damage and oncogenic transformation [7]. RIBE signals are transmitted *via* soluble mediators secreted into the extracellular space and *via* gap-junction intercellular communication [8]. RIBE has been linked to numerous hallmarks of cancer experimentally [9], however the majority of RIBE studies have been performed in *in vitro* models, with limited evidence using *ex vivo* human tissue.

Mitochondria are integral to RIBE induction and appear to have a dual role in the manifestation of RIBE. Cells deficient in mitochondrial DNA are unable to produce a bystander signal, highlighting the role of this organelle in RIBE [10]. Furthermore, inhibition of mitochondrial function inhibits RIBE signalling and response in human lymphoblastoid cells [11]. Previous work by Gorman et al. demonstrated that RIBE induction in an *ex vivo* model of CRC induced random mitochondrial point mutations in bystander cells [12]. Dissecting the role of mitochondrial metabolism and changes in the metabolome during RIBE has not yet been investigated.

Altered metabolism and mitochondrial function are important players in the response to radiation, where radioresistant oesophageal adenocarcinoma (OAC) cells show altered metabolism and changes in mitochondrial morphology [13]. Moreover, in patient tumours, higher oxidative phosphorylation (OXPHOS) is associated with radiation treatment resistance in OAC [13]. Furthermore, exposure of OAC cells to adipose conditioned media from viscerally obese OAC patients altered mitochondrial function and induced a metabolic shift in OAC cells, indicating that obesity modulates the behaviour of mitochondria in cancer cells [14].

To date, much radiation bystander research has been conducted *in vitro*, with some *in vivo* models in more recent years. To the best of our knowledge this is the first study to investigate RIBE induction in an *ex vivo* model of rectal cancer. We investigated the effect of *ex vivo* RIBE induction using rectal cancer and normal rectal tissue on bystander cellular metabolism, mitochondrial function and metabolomic profiling of the normal and cancer tissue microenvironments to determine if the bystander effects differed between normal and malignant rectal tissue. We hypothesised that *ex vivo* RIBE induction would induce metabolic alterations and alter mitochondrial function and these would differ between normal and malignant tissue. We also hypothesised that obesity status would be associated with metabolic perturbations in bystander cells.

## Methods

### Patient recruitment

Patients undergoing lower gastrointestinal investigations were prospectively recruited to this study between January 2018 and November 2018. Ethical approval was granted for the entire study by the St. James's Hospital/AMNCH Research Ethics Committee (Ref 2011/43/02) and the study was conducted in line with the Declaration of Helsinki and data protection regulations. All patients provided informed consent for their participation in this study. Biopsies were obtained from treatment-naïve rectal cancer patients and normal (non-cancer) control patients at diagnostic endoscopy. Clinical data was obtained from patient healthcare records.

The human rectal adenocarcinoma SW837 cell line purchased from European Collection of Authenticated Cell Culture (ECACC) was utilised for all bystander experiments in this study. This is a grade IV adenocarcinoma cell line obtained from a 53-year old male. The cells are p53 mutant with a C->T mutation in codon 248. Cells were cultured in Leibovitz's L-15 medium supplemented with 10% FBS and 1% penicillin-streptomycin.

### Generation of tumour conditioned media and normal conditioned media

Tumour conditioned media (TCM) and normal conditioned media (NCM) were generated by gently washing fresh biopsies four times in PBS supplemented with 1% penicillin-streptomycin, 1% Fungizone (amphotericin B) and 0.1% gentamicin. Each biopsy was then placed in 1 ml of M199 media supplemented with 10% FBS, 1% penicillin-streptomycin, 1% Fungizone, 0.1% gentamicin and 1 μg/ml insulin. Biopsies were incubated for 80 min at 37 °C and 5% CO_2_. Following 80-min incubation, biopsies were either mock-irradiated (0Gy) or irradiated with 1.8Gy radiation using an XStrahl RS225 x-irradiator at a dose rate of 1.73Gy/min (195 kV, 15 mA) [XSTRAHL, Surrey, UK] and incubated for 24 h at 37 °C and 5% CO_2_. The media was then harvested and stored in 2 ml cryotubes at −80 °C until required. The matched cultured biopsy tissues were snap-frozen in liquid nitrogen and stored at −80 °C.

For the purposes of all bystander experiments, a 1:1 dilution of TCM/NCM in M199 was used for all assays. This dilution was chosen based on optimisation experiments using TCM/NCM on SW837 cells with no differences being observed between the neat use of TCM/NCM and a 1:1 dilution of TCM/NCM with M199.

### Assessment of bystander metabolic profiles using Seahorse technology

SW837 cells were seeded in duplicate at a density of 30,000 cells per well in complete RPMI in a 24 well XFe24 cell culture microplate for 24 h [Agilent Technologies, Santa Clara, CA, USA] and then treated with NCM or TCM from the human normal or rectal cancer *ex vivo* explants respectively for 24 h. Following 24-h treatment, Oxygen Consumption Rate (OCR) and Extracellular Acidification Rate (ECAR) were measured using a Seahorse Biosciences XFe24 Extracellular Flux Analyser [Agilent Technologies, Santa Clara, CA, USA] as previously described [15].

### Assessment of reactive oxygen species and mitochondrial membrane potential levels in bystander cells

Reactive oxygen species (ROS) was assessed using the fluorescent probe 2′-7′-dichlorofluorescien (2′-7′-DCF). Mitochondrial membrane potential (mtMP) was assessed using the fluorescent probe Rhodamine 123. SW837 cells were seeded at 30,000 cells per well in a 96-well plate for 24 h. Following 24-h incubation, cells were treated with 100 μl of TCM or NCM for 24 h. ROS was assessed by adding 50 μl of 2,7-DCF (5 μM) in PBS-Mg and mtMP was assessed by adding 50 μl of Rhodamine 123 (5 μM) in PBS-Mg as described previously [15]. All seahorse data and mitochondrial function assay data was normalised to cell number using the crystal violet assay as previously described [15].

### Evaluation of changes in the metabolome of normal and rectal cancer *ex vivo* explants in response to radiation using metabolomics

A total of 250 μl deuterium oxide (D_2_O) and 10 μl sodium trimethyl [2,2,3,3-^2^H_4_] propionate (TSP) (0.005 g/ml) were added to each 300 μl TCM, NCM and control sample. Spectra were acquired on a 600-MHz Varian nuclear magnetic resonance (^1^HNMR) spectrometer [Varian Limited, Oxford, United Kingdom] by using the Carr-Purcell-Meiboom-Gill (CPMG) pulse sequence at 25 °C. Spectra were acquired with 16,384 data points and 256 scans. Water suppression was achieved during the relaxation delay (3.0 s). ^1^HNMR spectra were referenced to TSP and were processed manually with Chenomx NMR Suite [Version 8.3; Chenomx Edmonton, Canada] by using a line broadening of 0.2 Hz, and all spectra were phase and baseline corrected. Using 0.005 ppm bins, spectra were converted into 1360 spectral regions. The water region (4.3 to 5.5 ppm) was excluded, and data normalised to the total area of the spectral integral. Identification of discriminating metabolites was also performed using Chenomx NMR Suite. Metabolites were profiled in the Chenomx NMR Suite.

### Body composition analysis by computed tomography

Computed tomography (CT) scans were obtained at diagnosis using a Discovery ST CT scanner [GE Healthcare, Little Chalfont, UK]. Images were analysed at L3 and the cross-sectional area in cm^2^ of the various tissue compartments was determined using TomoVision Sliceomatic version 5.0 software [TomoVision, Montreal, Canada], applying an automated algorithm utilizing CT Hounsfield unit threshold of −29 to 150 for skeletal muscle and −50 to −150 for adipose tissue [[16], [17], [18]].

Visceral fat area (VFA) was calculated by a radiologist using a previously standardised and validated technique, and patients with a VFA greater than 163.8cm^2^ (males) and 80.1cm^2^ (females) were classified as obese [17].

### Statistical analysis

GraphPad Prism 5 software was used to perform statistical analysis. All data is expressed as mean ± SEM. Specific statistical test indicated in each figure legend. Correlations were performed using Pearson correlation analysis. Statistical significance considered at *p* ≤ 0.05.

Multivariate statistical analysis was performed for the metabolomics data analyses with Simca- P+ software (Version 13.0.3; Umetrics, Umea°, Sweden). Unsupervised principal component analysis (PCA) was applied to the metabolomics data to explore any trends in the data for the various comparison groups, followed by partial least square discriminant analysis (PLS-DA) and orthogonal partial least squares discriminant analysis (OPLS-DA). The goodness-of-fit parameter (R^2^) and the predictive ability parameter (Q^2^) was used to describe the quality of all models. An independent *t*-test was performed on discriminating metabolite areas between comparison groups using IBM SPSS Statistics version 24. Statistical significance was considered if *p* ≤ 0.05.

## Results

### Patient characteristics

All clinical data for rectal cancer and control patients is shown in Table 1.

**Table 1:** Patient characteristics.

			Percent (%)
Age (rectal cancer patients)	Mean ± SD	65.16 ± 10.14	
	Range	53–89	
Age (control patients)	Mean ± SD	58.75 ± 11.42	
	Range	44–76	
Gender (rectal cancer patients)	Male (n)	8	66.67
	Female (n)	4	33.33
Gender (control patients)	Male (n)	3	37.5
	Female (n)	5	62.5
Obesity status (visceral fat area)	Obese	8	66.67
	Non obese	4	33.33
Histology	Adenocarcinoma (n)	12	100
Stage of differentiation	Moderate (n)	12	100
T stage	T1 (n)	1	8.33
	T2 (n)	2	16.67
	T3 (n)	8	66.67
	T4 (n)	1	8.33
N stage	N0 (n)	6	50
	N1 (n)	5	41.67
	N2 (n)	1	8.33
M stage	M0 (n)	11	91.67
	M1 (n)	1	8.33
Neoadjuvant CRT	Received neo-CRT (n)	6	50
Neoadjuvant CT	Received neo-CT only (n)	1	8.33
Neoadjuvant RT	Received neo-RT only (n)	1	8.33
Surgery^a^	Received surgery (n)	11	91.67
TRS^b^	0 (n)	2	28.57
	1 (n)	3	42.85
	2 (n)	1	14.28
	3 (n)	1	14.28

Abbreviations; CRT; chemoradiotherapy, CT; chemotherapy, RT; radiotherapy, TRS; tumour regression score.

a One patient unsuitable for surgery.

b TRS available for 7 patients, percent (%) expressed as percent (%) of those with TRS score.

### RIBE induction *ex vivo* induces significant changes in oxidative phosphorylation and glycolysis in bystander rectal cancer cells

**Fig. 1:**
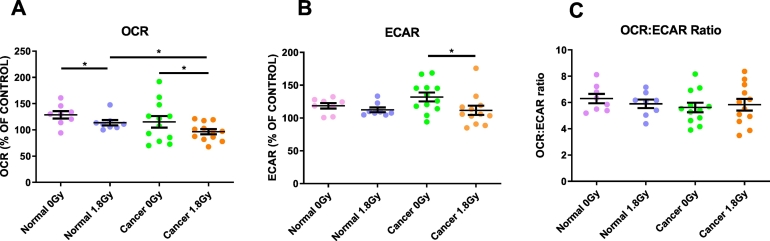
Effect of *ex vivo* RIBE induction in normal rectal tissue and rectal cancer tissue on OCR, ECAR and OCR:ECAR ratio in bystander SW837 cells. (A) *Ex vivo* RIBE induction in both normal rectal and rectal cancer tissue causes significant reductions in OCR in bystander SW837 cells, when compared to cells exposed to the unirradiated tissue secretome. (B) ECAR is significantly reduced in bystander SW837 cells treated with TCM from irradiated rectal cancer tissue compared to mock-irradiated rectal cancer tissue. (C) OCR:ECAR ratio is not significantly different in bystander SW837 cells treated with NCM or TCM from irradiated *ex vivo* explants compared to their unirradiated controls. All data expressed as mean ± SEM. Statistical analysis was performed by paired *t*-test for 0Gy *vs* 1.8Gy when comparing the same tissue and unpaired *t*-test when comparing different tissue. n=8 normal, n=12 cancer, *p < 0.05.

Following 24-h treatment with either TCM or NCM, OCR, a measure of OXPHOS, and ECAR, a measure of glycolysis, were assessed. Significant reductions in OCR were observed in bystander cells exposed to NCM from irradiated normal rectal tissue, when compared to those exposed to the NCM from the mock-irradiated normal rectal tissue (*p* = 0.01). Similarly, there was a significant reduction in OCR in SW837 bystander cells exposed to TCM from irradiated rectal cancer tissue compared to those exposed to TCM from mock-irradiated rectal cancer tissue (*p* = 0.03) (Fig. 1A). Bystander SW837 rectal cancer cells treated for 24 h with TCM from irradiated rectal cancer biopsies, showed a significant reduction in ECAR, when compared to SW837 cells treated with TCM from mock-irradiated biopsies (*p* = 0.014) (Fig. 1B).

OCR:ECAR ratio was not significantly different in SW837 cells treated with NCM from mock-irradiated or irradiated normal rectal tissue (*p* = 0.25), or cancer tissue (*p* = 0.38). No difference was observed in OCR:ECAR ratio between SW837 cells treated with NCM or TCM (*p* = 0.21) (Fig. 1C).

This has demonstrated for the first time, real-time metabolic alterations in bystander cells following exposure to TCM and NCM from human rectal biopsies. Moreover, we have demonstrated a differential response in bystander cells exposed to TCM from malignant rectal tissue and NCM from normal rectal tissue, with alterations in glycolysis only observed in bystander cells exposed to TCM from malignant tissue.

### RIBE induction *ex vivo* in both normal rectal tissue and rectal cancer tissue causes significant reductions in basal respiration, ATP production and maximal respiration in bystander SW837 cells

**Fig. 2:**
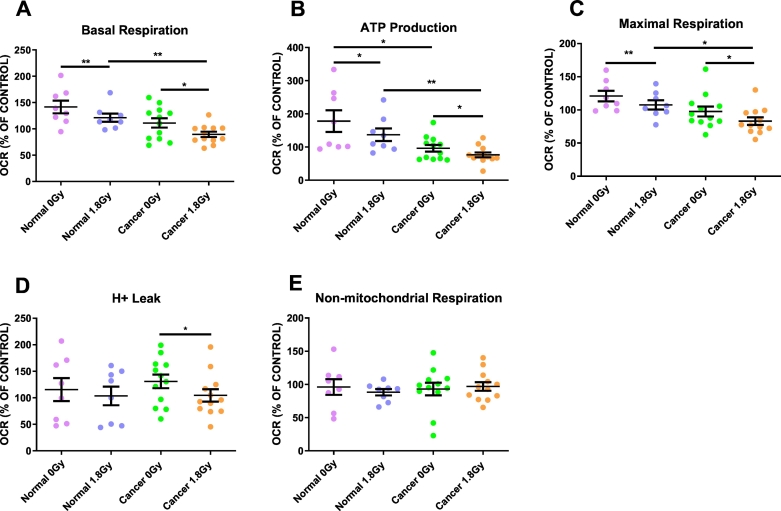
Effect of *ex vivo* RIBE induction on SW837 bystander basal respiration, ATP production, maximal respiration, proton leak and non-mitochondrial respiration. (A) RIBE induction in *ex vivo* normal rectal and rectal cancer tissue induces significant reductions in basal respiration in bystander SW837 cells, when compared to cells exposed to the unirradiated tissue secretome. (B) ATP production is reduced in bystander SW837 cells treated with NCM and TCM from irradiated normal and rectal cancer tissue, respectively. ATP production is significantly reduced in bystander cells treated with TCM from the cancer secretome compared to NCM from the normal rectal secretome. (C) *Ex vivo* RIBE induction in both normal rectal and rectal cancer tissue significantly reduces maximal respiration in bystander SW837 rectal cancer cells. (D) Proton leak was significantly lower in bystander cells treated with TCM from the irradiated rectal cancer secretome (E) No significant differences were observed in non-mitochondrial respiration. All data expressed as mean ± SEM. Statistical analysis was performed by paired *t*-test for 0Gy *vs* 1.8Gy when comparing the same tissue and unpaired *t*-test when comparing different tissue. n=8 normal, n=12 cancer, **p < 0.01, *p < 0.05.

Basal respiration was significantly lower in SW837 cells exposed to NCM from irradiated normal rectal tissue, when compared to SW837 cells exposed to NCM from mock-irradiated normal rectal tissue (*p* = 0.009). Similarly, basal respiration was significantly lower in SW837 cells treated with TCM from irradiated rectal cancer tissue, when compared to mock-irradiated rectal cancer tissue (*p* = 0.029) (Fig. 2A).

A significant reduction in ATP production was observed in SW837 bystander cells treated with NCM from irradiated biopsies, when compared to SW837 cells exposed to NCM from mock-irradiated biopsies (*p* = 0.039). ATP production was significantly lower in SW837 cells treated with TCM from irradiated rectal cancer biopsies, when compared to mock-irradiated rectal cancer biopsies (*p* = 0.043). Significantly lower levels of ATP production were observed in SW837 cells treated with TCM from mock-irradiated biopsies, when compared to those treated with NCM from mock-irradiated biopsies (*p* = 0.011) (Fig. 2B).

Maximal respiration was significantly reduced in SW837 cells treated with NCM from irradiated normal rectal tissue, when compared to those treated with NCM from mock-irradiated tissue (*p* = 0.009). SW837 cells exposed to TCM from irradiated rectal cancer tissue showed a significant reduction in maximal respiration, when compared to SW837 cells treated with TCM from mock-irradiated rectal cancer tissue (*p* = 0.017) (Fig. 2C).

There was no significant difference in non-mitochondrial respiration between SW837 cells treated with NCM from either mock-irradiated or irradiated normal rectal tissue (*p* = 0.59) or cancer tissue (*p* = 0.60) (Fig. 2E). Proton leak was significantly reduced in SW837 cells treated with TCM from irradiated rectal cancer biopsies compared to mock-irradiated rectal cancer biopsies (*p* = 0.02). The difference in proton leak between SW837 cells treated with NCM from mock-irradiated or irradiated normal rectal tissue failed to reach statistical significance (*p* = 0.09) (Fig. 2D).

To date, the majority of bystander work has utilised *in vitro* models, however, our work recapitulates the complexity of the tumour and normal microenvironment encompassing the 3D architecture and multiple cell types. This is the first study to profile real-time metabolic changes in cells following exposure to culture media from human rectal cancer and normal rectal biopsies.

### Rectal cancer secretome induces a significant increase in reactive oxygen species levels compared to the normal rectal secretome in bystander SW837 cells

**Fig. 3:**
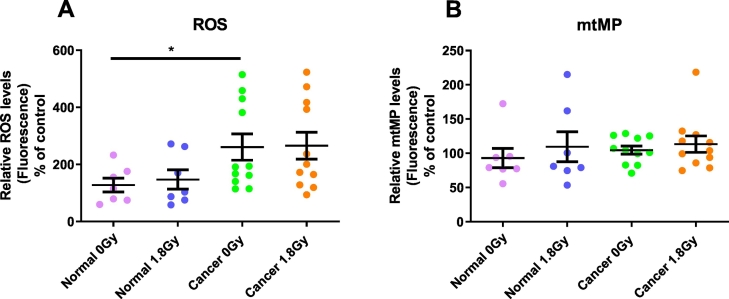
The cancer secretome increases ROS levels in bystander SW837 rectal cancer cells. (A) ROS levels are significantly higher in bystander SW837 cells treated with TCM from rectal cancer tissue (n=11) compared to cells treated with NCM from normal rectal tissue (n=7). (B) No changes in mtMP were detected in bystander cells exposed to all *ex vivo* conditions. All data expressed as mean ± SEM. Statistical analysis was performed by paired *t*-test for 0Gy *vs* 1.8Gy when comparing the same tissue and unpaired *t*-test when comparing different tissue. *p < 0.05.

ROS levels were significantly higher in SW837 cells following TCM treatment from mock-irradiated rectal cancer tissue, when compared to NCM from mock-irradiated normal rectal tissue (*p* = 0.04). The difference in ROS levels in SW837 cells treated with TCM from irradiated rectal cancer tissue, when compared to SW837 cells treated with NCM from irradiated normal rectal tissue was not significant (*p* = 0.08) nor did ROS levels change between SW837 cells treated with NCM from either irradiated or mock-irradiated normal rectal tissue (*p* = 0.21), or between bystander cells treated with TCM from irradiated or mock-irradiated rectal cancer tissue (*p* = 0.54) (Fig. 3A). No changes in mtMP were detected in bystander cells exposed to all *ex vivo* conditions (Fig. 3B).

These results suggest that the rectal cancer microenvironment induces higher levels of oxidative stress compared to the normal rectal microenvironment, but that RIBE *per se* does not alter mitochondrial function following 24 h exposure to TCM or NCM.

### Metabolomic profiling of NCM and TCM from mock-irradiated and irradiated normal rectal and rectal cancer tissue

**Fig. 4:**
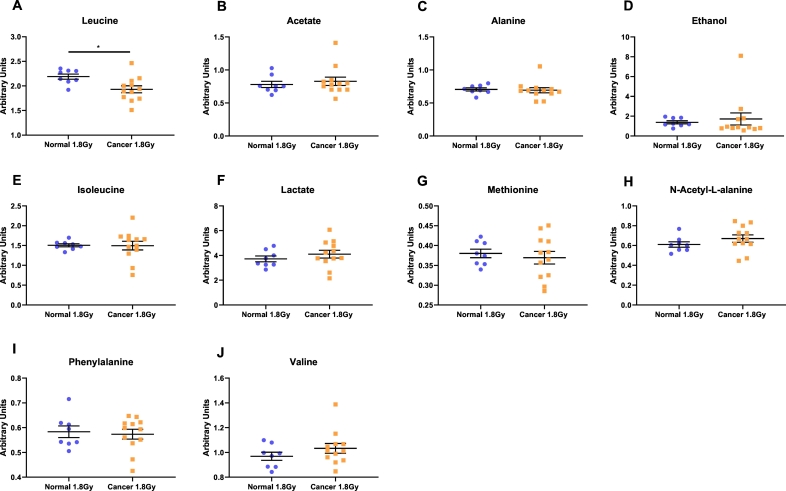
Leucine levels are reduced in the secretome of irradiated rectal cancer tissue compared to irradiated normal rectal tissue. (A) Leucine levels are significantly reduced in the secretome of irradiated rectal cancer tissue compared to irradiated normal rectal tissue. There was no significant difference in (B) acetate (C) alanine (D) ethanol (E) isoleucine (F) lactate (G) methionine (H) *N*-Acetyl-L-alanine (I) phenylalanine or (J) valine levels between the irradiated rectal cancer secretome and the irradiated normal rectal secretome. All data expressed as mean ± SEM. Statistical analysis was performed by unpaired *t*-test. *p < 0.05. n=8 normal, n=12 cancer.

To better understand what metabolites may be driving these observed mitochondrial and metabolic bystander events, metabolomic screening was performed. A weak PLS-DA model was obtained for TCM from irradiated rectal cancer tissue *vs* NCM from irradiated normal rectal tissue (R^2^x = 0.591, Q2 = 0.153), indicating no major changes in the global metabolomic profile. No other changes in the global metabolomic profile were observed when comparing the mock-irradiated and irradiated samples. However, profiling of specific metabolites revealed that the amino acid leucine was significantly different between NCM from irradiated normal rectal tissue, when compared to TCM from irradiated rectal cancer tissue (*p* = 0.041) (Fig. 4A). Leucine levels were reduced in the TCM from irradiated rectal cancer tissue compared to NCM from irradiated normal rectal tissue (Supp. Table 1). There were no significant changes in levels of acetate, alanine, ethanol, isoleucine, lactate, methionine, *N*-Acetyl-L-alanine, phenylalanine or valine (Fig. 4B–J).

### Linking mitochondrial function to energy metabolism

In order to determine if there was any relationship between altered energy metabolism and mitochondrial function, we correlated ROS and mtMP levels with metabolic readouts obtained from the Seahorse. There was a significant inverse correlation observed between ROS levels and OCR (R = −0.8101, *p* = 0.002), basal respiration (R = −0.8745, *p* = 0.0004) and maximal respiration (R = −0.6696, *p* = 0.02) in bystander cells treated with TCM from mock-irradiated rectal cancer tissue. Similarly, ROS correlated inversely with OCR (R = −0.8105, *p* = 0.02), basal respiration (R = −0.8194, *p* = 0.02), maximal respiration (R = −0.8101, *p* = 0.02) and ATP production (R= −0.7718, *p* = 0.04) in NCM from mock-irradiated normal rectal tissue. OCR:ECAR ratio significantly positively correlated with ROS levels in bystander cells treated with the secretome of both mock-irradiated (R = 0.8136, *p* = 0.002) and irradiated (R = 0.9064, *p* = 0.0001) rectal cancer tissue. Proton leak was inversely correlated with ROS levels in cells treated with the rectal cancer secretome of mock-irradiated tissue (R = −0.7988, *p* = 0.003). An inverse correlation was observed between ROS and maximal respiration (R= −0.7648, *p* = 0.04) and proton leak (R=−0.7554, *p* = 0.04) in the secretome of irradiated normal rectal tissue and a positive correlation between ROS and non-mitochondrial respiration was observed in the same tissue (R = 0.8315, *p* = 0.02). There was an inverse correlation between OCR and ROS in bystander cells treated with TCM from irradiated rectal cancer tissue (R = −0.7223, *p* = 0.01) (Table 2, Table 3).

**Table 2:** Correlation between ROS levels and metabolic parameters in normal tissue.

NORMAL 0GY					NORMAL 1.8GY				
FACTOR (% of control)	R value	p-value	Significance	n	FACTOR (% of control)	R value	p-value	Significance	n
OCR	-0.8105	0.02	***, ***	7	Non-mitochondrial respiration	0.8315	0.02	*	7
Basal respiration	-0.8194	0.02	*	7	Maximal respiration	-0.7648	0.04	*	7
ATP production	-0.7718	0.04	*	7	H+ leak	-0.7554	0.04	*	7
Maximal respiration	-0.8101	0.02	*	7					

* *p* < 0.05,

** *p* <0.01,

*** *p <* 0.001

**Table 3:** Correlation between ROS levels and metabolic parameters in cancer tissue.

CANCER 0GY					CANCER 1.8GY				
FACTOR (% of control)	R value	p-value	Significance	n	FACTOR (% of control)	R value	p-value	Significance	n
OCR	-0.8101	0.002	**	11	OCR	-0.7223	0.01	*	11
Basal Respiration	-0.8745	0.0004	***	11	OCR:ECAR Ratio	0.9064	0.0001	***	11
H+ leak	-0.7988	0.003	**	11					
Maximal respiration	-0.6696	0.02	*	11					
OCR:ECAR Ratio	0.8136	0.002	**	11					
mtMP	-0.7229	0.01	*	11					

* *p* < 0.05,

** *p* <0.01,

*** *p <* 0.001

There was a significant inverse correlation between ROS and mtMP in bystander SW837 cells treated with TCM from mock-irradiated rectal cancer tissue (R = −0.7229, *p* = 0.01). This result was expected since increases in ROS are known to cause depolarisation of mtMP which in turn triggers mitophagy [19]. There were no significant correlations between ROS and mtMP in bystander cells treated with TCM from irradiated rectal cancer tissue or NCM from either irradiated or mock-irradiated normal rectal tissue (Table 3).

mtMP correlated with OCR (R = 0.7038, *p* = 0.01), basal respiration (R = 0.8013, *p* = 0.003), H+ leak (R = 0.7147, *p* = 0.01) and maximal respiration (R = 0.6305, *p* = 0.03) in bystander cells treated with TCM from mock-irradiated rectal cancer tissue. Similarly, there was a positive correlation between mtMP and OCR (R = 0.6343, *p* = 0.03) and maximal respiration (R = 0.6817, *p* = 0.02) in bystander cells treated with TCM from irradiated rectal cancer tissue (Table 4).

**Table 4:** Correlation between mtMP levels and metabolic parameters in SW837 cells treated with TCM from cancer tissue.

Cancer 0GY					Cancer 1.8GY				
FACTOR (% of control)	R value	p-value	Significance	n	FACTOR (% of control)	R value	p-value	Significance	n
OCR	0.7038	0.01	*	11	OCR	0.6343	0.03	*	11
Basal respiration	0.8013	0.003	*****	11	Maximal respiration	0.6817	0.02	*	11
H+ leak	0.7147	0.01	*	11					
Maximal respiration	0.6305	0.03	*	11					

* *p* < 0.05,

** *p* <0.01,

*** *p <* 0.001

We analysed all metabolic and mitochondrial function data according to T stage and N stage and no significant differences emerged between any metabolic readout or mitochondrial function parameter in either the unirradiated or irradiated malignant tissue.

### Linking bystander cell metabolism to metabolite levels in the secretome of normal and cancer tissue

In order to determine if there was any relationship between metabolite levels in the secretome of normal rectal tissue and rectal cancer tissue both pre- and post-radiation and bystander cellular metabolism, we conducted correlation analysis to identify any relationship between these factors. We found a significant correlation between OCR in bystander cells and methionine levels in the secretome of mock-irradiated normal rectal tissue (R = 0.7139, *p* = 0.04). We also found correlations between non-mitochondrial respiration and alanine (R = 0.7452, *p* = 0.03) and phenylalanine (R = 0.8073, *p* = 0.01) levels in the secretome of mock-irradiated normal rectal tissue. Proton leak correlated inversely with phenylalanine (R = −0.7501, *p* = 0.03) OCR:ECAR ratio in bystander cells correlated with levels of isoleucine (R = 0.7657, *p* = 0.02), *N*-Acetyl-L-alanine (R = 0.8154, *p* = 0.01), phenylalanine (R = 0.7542, *p* = 0.03), valine (R = 0.8357, *p* = 0.009) and alanine (R = 0.7874, *p* = 0.02) in mock-irradiated normal rectal tissue. In bystander cells exposed to NCM from irradiated normal rectal tissue, OCR:ECAR ratio correlated inversely with both acetate (R = −0.7736, *p* = 0.02) and lactate (R=−0.7161, *p* = 0.04) (Table 5).

**Table 5:** Correlation between metabolic parameters in bystander cells and metabolites in the secretome of normal rectal tissue.

NORMAL 0GY					NORMAL 1.8GY				
FACTOR (% of control)	Metabolite	R value	p-value	n	FACTOR (% of control)	Metabolite	R value	p-value	n
OCR	Methionine	0.7139	0.04	8	OCR:ECAR Ratio	Acetate	-0.7736	0.02	8
Non mitochondrial respiration	Alanine	0.7452	0.03	8	OCR:ECAR Ratio	Lactate	-0.7161	0.04	8
Non mitochondrial respiration	Phenylalanine	0.8073	0.01	8					
H+ leak	Phenylalanine	-0.7501	0.03	8					
OCR:ECAR Ratio	Isoleucine	0.7657	0.02	8					
OCR:ECAR Ratio	N-Acetyl L-alanine	0.8154	0.01	8					
OCR:ECAR Ratio	Phenylalanine	0.7542	0.03	8					
OCR:ECAR Ratio	Valine	0.8357	0.009	8					
OCR:ECAR Ratio	Alanine	0.7874	0.02	8					

In bystander cells treated with TCM from mock-irradiated rectal cancer tissue, non-mitochondrial respiration correlated inversely with ethanol levels in the secretome of mock-irradiated rectal cancer tissue (R = −0.7399, *p* = 0.005). In bystander cells treated with TCM from irradiated rectal cancer tissue, OCR correlated with methionine levels in the secretome of this TCM (R = 0.5812, *p* = 0.04). Basal respiration in bystander cells treated with TCM from irradiated rectal cancer tissue correlated with methionine (R = 0.6561, *p* = 0.02) and *N*-Acetyl-L-alanine (R = 0.5760, *p* = 0.05). Maximal respiration also correlated with methionine (R = 0.7315, *p* = 0.006). Interestingly, OCR:ECAR ratio in bystander cells treated with TCM from irradiated rectal cancer tissue correlated inversely with methionine (R = −0.7017, *p* = 0.01), *N*-Acetyl-L-alanine (R = −0.7165, *p* = 0.008) and alanine (R = −0.7045, *p* = 0.01). (Table 6). This is a differential response to the cells treated with NCM from irradiated normal rectal tissue.

**Table 6:** Correlation between metabolic parameters in bystander cells and metabolites in the secretome of rectal cancer tissue.

CANCER 0GY					CANCER 1.8GY				
FACTOR (% of control)	Metabolite	R value	p-value	n	FACTOR (% of control)	Metabolite	R value	p-value	n
Non-mitochondrial respiration	Ethanol	-0.7399	0.005	12	OCR	Methionine	0.5812	0.04	12
					Basal Respiration	Methionine	0.6561	0.02	12
					Basal Respiration	N-Acetyl-L-alanine	0.5760	0.05	12
					Maximal Respiration	Methionine	0.7315	0.006	12
					OCR:ECAR Ratio	Methionine	-0.7017	0.01	12
					OCR:ECAR Ratio	N-Acetyl-L-alanine	-0.7165	0.008	12
					OCR:ECAR Ratio	Alanine	-0.7045	0.01	12

This highlights the differential relationship between altered metabolites and metabolic parameters in bystander cells treated with NCM and TCM depending on whether or not the tissue was irradiated.

### Linking body composition analysis to metabolism and mitochondrial function in bystander cells and secreted metabolite levels in the secretome of rectal cancer tissue

It has been reported that overweight or obese rectal cancer patients have a poorer response to neoadjuvant treatment compared to their counterparts of a normal weight [20,21] as well as worse local control following neoadjuvant treatment and surgery [22]. We sought to understand the relationship between body composition and bystander cellular response to TCM from both irradiated and mock-irradiated rectal cancer biopsies.

We found a significant correlation between ECAR in bystander SW837 cells treated with TCM from irradiated rectal cancer biopsies and VFA (R = 0,6446, *p* = 0.02) and skeletal muscle mass (R = 0.8034, *p* = 0.001). There was also a significant correlation between ATP production in bystander SW837 cells treated with TCM from irradiated rectal cancer biopsies and VFA (R = 0.6204, *p* = 0.03) (Table 7). Of note, these observations only occurred in the bystander cells treated with TCM from irradiated rectal cancer biopsies, perhaps suggesting that obesity status may influence the behaviour of bystander cells following radiation. It is well documented that upregulation of glycolysis is associated with a radioresistant phenotype [[23], [24], [25], [26]] and induces DNA repair pathways [27]. Therefore, it may be possible that factors secreted from the TME of obese individuals may alter the metabolism of tumour cells and facilitate a more radioresistant phenotype.

**Table 7:** Correlation between metabolic parameters and body composition analysis in bystander cells treated with TCM from irradiated rectal cancer biopsies i.e. Cancer 1.8Gy.

Body composition parameter	FACTOR (% of control)	R value	p-value	n
VFA	ECAR	0.6446	0.02	12
VFA	ATP production	0.6204	0.03	12
Skeletal muscle	ECAR	0.8034	0.001	12

Abbreviations: VFA; visceral fat area, ECAR; extracellular acidification rate (glycolysis)

We found significant correlations between VFA and leucine (R = 0.6395, *p* = 0.02) and ethanol (R = 0.5972, *p* = 0.04) and intermuscular fat and ethanol (R = 0.6483, *p* = 0.02) levels in the TCM from mock-irradiated rectal cancer tissue (Table 8). This data suggests that obesity status may influence the local milieu of rectal cancer tissue by altering metabolite levels in the TME.

**Table 8:** Correlation between body composition parameters and metabolites in the secretome of unirradiated rectal cancer tissue (Cancer 0Gy).

Body composition parameter	Metabolite	R value	*p*-Value	n
VFA	Leucine	0.6395	0.02	12
VFA	Ethanol	0.5972	0.04	12
Intermuscular fat	Ethanol	0.6483	0.02	12

## Discussion

Using a novel human *ex vivo* explant model of rectal cancer and normal rectal tissue, we have shown for the first time that RIBE induction *ex vivo* causes significant alterations in bystander cellular energy metabolism. We have demonstrated that metabolite profiles, specifically leucine concentrations, differed in the secretome between irradiated rectal cancer tissue and irradiated normal rectal tissue, with leucine levels being reduced in the irradiated cancer secretome compared to the irradiated normal secretome. We have demonstrated significant differences in ROS but not mtMP in bystander cells exposed to the rectal cancer, when compared to the normal rectal secretome. We found significant correlations between VFA and ECAR and ATP production in bystander cells treated with TCM from irradiated rectal cancer biopsies, while ECAR in these bystander cells also correlated with skeletal muscle mass.

The majority of bystander work to date has employed *in vitro* models and 3D tissue models [28], with others employing *in vivo* models such as fish [29,30] and rodents [31]. Our work in this study explores the complexity of the tumour microenvironment (TME) and the normal rectal microenvironment, using *ex vivo* explants. Importantly, these whole biopsy explants recapitulate the microenvironment of both normal and malignant rectal tissue. This allows us to capture the effect of radiation on all cell types within these microenvironments and elucidate if the response of bystander cancer cells to RIBE signals differs between normal and malignant tissue.

We have shown that *ex vivo* RIBE induction in both normal rectal tissue and rectal cancer tissue causes significant reductions in OXPHOS in bystander SW837 rectal cancer cells. Le et al. have previously shown reduced complex I activity in the mitochondria of bystander HCT116 CRC cells exposed to RIBE-induced biophoton signalling [32]. In our study, ATP production and maximal respiration were also significantly reduced following RIBE in bystander cells, which is in line with reduced OCR in these cells. Interestingly, a reduction in glycolysis was only observed following RIBE induction in malignant tissue. A more pronounced effect on glycolysis was expected in bystander cells exposed to TCM since malignant tissue is known to have a greater tendency to employ aerobic glycolysis compared to normal tissue [33]. To date, this is the first study to investigate the effect of *ex vivo* RIBE induction in rectal tissue on real-time cellular metabolism using Seahorse technology.

ROS are well-recognised participants in RIBE events [10,34,35]. Mitochondria are an important source of endogenous ROS and also, traversal of the nucleus with radiation was not required to exert RIBE effects [36] and antioxidants can abrogate RIBE [37]. Given the observed changes in mitochondrial metabolism in our study combined with the well-recognised role for the mitochondria in RIBE, we examined the effect of *ex vivo* RIBE induction on bystander rectal cancer cellular mitochondrial function, specifically ROS production and mtMP.

RIBE-induction *ex vivo* did not alter the mtMP or ROS levels in bystander SW837 rectal cancer cells. Similar results were obtained by Gorman et al. in bystander SW480 cells. However, the authors did report significantly reduced levels of mtMP in bystander cells exposed to TCM and NCM from irradiated CRC tissue and irradiated normal adjacent tissue compared to their unirradiated controls [12]. ROS are known to be short-lived molecules and studies have shown changes in ROS and mtMP levels as early as 4 h and 12 h, following exposure to conditioned media but levels of both ROS and mtMP returned to normal at 24 h [38]. It may be possible in our study that ROS and mtMP returned to baseline levels following 24-h treatment with TCM and NCM from irradiated tissue.

The rectal cancer secretome caused significant elevations in ROS levels in bystander SW837 cells compared to bystander cells exposed to the normal rectal secretome. It is a well-documented phenomenon that cancer cells have significantly higher levels of ROS compared to normal cells, owing to oncogenic transformation, including altered cellular metabolism, genetic mutation and a pro-tumorigenic microenvironment [39]. Elevated ROS promotes oncogenic DNA mutations and may activate oncogenic signalling pathways [40]. ROS may also be capable of initiating oncogenic signalling pathways such as EGFR [41] and MAPK [42] signalling pathways. Therefore, it is unsurprising that ROS levels were higher in bystander cells exposed to the rectal cancer microenvironment compared to the normal rectal microenvironment.

To better understand what metabolites may be driving these mitochondrial and metabolic bystander events, metabolomic screening was performed. PCA analysis of ^1^HNMR spectra obtained from screening the TCM and NCM from both irradiated and mock-irradiated rectal cancer and normal rectal tissue allowed separation of the irradiated normal and the irradiated cancer tissue secretome. Further analysis of the metabolite profile of the TCM and NCM indicated reduced levels of leucine in the secretome of the irradiated rectal cancer tissue compared to the irradiated normal rectal tissue. The literature supports this observation. Arenas et al. have shown that serum leucine levels are significantly lower in breast cancer patients compared to control patients and following radiation therapy, leucine levels return to levels observed in control patients [43]. He et al. report higher levels of leucine within the pancreas of mice bearing pancreatic tumours compared to the pancreas of control mice [44]. Ching et al. found significantly lower levels of leucine in the plasma of cancer patients receiving chemotherapy [45]. The authors postulated that these observations were owing to enhanced uptake of leucine by cancer cells [[43], [44], [45]]. Since the metabolites in our study were not labelled, it was not possible to determine whether alterations in these metabolites were owing to altered secretion of metabolites by the tissue or altered uptake by cancer tissue. However, we believe it is likely owing to enhanced uptake of leucine by cancer cells, as has been reported in the literature. Following a full course of radiation therapy, Arenas et al. observed that leucine levels returned to levels observed in control patients. It is important to note that the changes in serum leucine concentrations post-radiotherapy were observed following a full treatment course of radiation therapy [43]. It may be possible that a single fraction of a clinically relevant dose of radiation, as used in our study may be insufficient to restore metabolite levels in the TME to that observed in non-cancerous tissue. Overall limited numbers of studies have investigated the difference in metabolites between normal and cancer tissue as well as the effect of radiation on metabolite levels. A limitation of our study is the number of participants, with 8 controls and 12 cancer patients; it may be possible that no difference in leucine levels observed between the unirradiated control samples and the unirradiated tumour samples is as a result of small numbers.

There was a significant inverse correlation between ROS and mtMP in bystander SW837 cells treated with TCM from mock-irradiated rectal cancer tissue. This result was expected since increases in ROS are known to cause depolarisation of mtMP which in turn triggers mitophagy [19]. We found significant inverse correlations between OCR and ROS levels in bystander SW837 rectal cancer cells treated with both NCM and TCM. This result was unexpected since previous work in our group has shown that ROS levels were higher in OAC cells with higher OCR [46]. However, since ROS are short-lived molecules it may be possible that higher ROS levels would be observed at earlier time-points, between 0 and 6 h.

Interestingly, we observed a significant correlation between ECAR and ATP production in bystander SW837 cells treated with TCM from irradiated rectal cancer biopsies and the patient's VFA. Of note, this observation only occurred in the bystander cells treated with TCM from irradiated rectal cancer biopsies, perhaps suggesting that obesity status may influence the behaviour of bystander cells following radiation. This is an interesting and clinically relevant result as it has been reported that overweight and obese patients have poorer responses to neoadjuvant treatment and worse outcomes in rectal cancer [[20], [21], [22]]. Moreover, when we correlated levels of metabolites in the secretome of mock-irradiated rectal cancer tissue we found significant correlations between VFA and leucine and ethanol. Also, intermuscular fat correlated with ethanol levels. This is an interesting observation and may suggest that obesity status may alter the TME by influencing the levels of metabolites. There were also correlations between metabolites in the microenvironment of both tumour and normal tissue pre- and post-radiation and bystander cellular metabolic parameters. Again, this suggests that altered metabolite levels cause alterations in cellular metabolism and the data suggests that obesity status impacts the levels of these metabolites.

It is well documented that upregulation of glycolysis is associated with a radioresistant phenotype [[23], [24], [25], [26]] and induces DNA repair pathways [27]. Moreover, Lynam-Lennon et al. have demonstrated an enhancement of glycolysis when OAC cells were exposed to adipose conditioned media from obese patients [14]. Similarly, elevated ATP levels are associated with enhanced radioresistance [46], while reduction of ATP levels have been associated with increased radiosensitivity [15,47]. While further elucidation of the mechanisms linking obesity with poorer response rates in rectal cancer is needed, this data offers insight into the effect of bystander signals from rectal cancer biopsies and how obesity modulates the bystander response.

To the best of our knowledge, this is the first study to investigate *ex vivo* RIBE induction in normal rectal tissue from healthy controls compared to rectal cancer tissue and its effect on bystander cellular metabolism and mitochondrial function. While the role of the mitochondria and ROS are well established in RIBE events *in vitro*, this novel study extends the understanding of the role of the mitochondria to RIBE events using a human *ex vivo* model of disease. Radiation is used in both the neoadjuvant and adjuvant setting in rectal cancer patients but pCR is only observed in a small subset of patients in the neoadjuvant setting. Further understanding the biological effects of radiation on unirradiated cells near the irradiated volume and how this relates to treatment response may reveal potential therapeutic targets to enhance radiosensitivity in the neoadjuvant setting.

The following is the supplementary data related to this article.

Supplementary Table 1 Table of metabolites discriminating NCM from irradiated normal rectal tissue compared to TCM from irradiated rectal cancer tissue. Supplementary Table 1

## CRediT authorship contribution statement

**Aisling B Heeran:** Conceptualisation, formal analysis, investigation, writing – original draft, visualisation, funding acquisition. **Helen P Berrigan:** Investigation, formal analysis, writing – review and editing. **Croí E. Buckley:** Investigation, writing – review and editing. **Heleena Moni Bottu:** Investigation, formal analysis, writing – review and editing. **Orla Prendiville:** Investigation, formal analysis, writing – review and editing. **Amy M. Buckley:** Resources, writing – review and editing. **Niamh Clarke:** Resources, writing – review and editing. **Noel E. Donlon:** Formal analysis, resources, writing – review and editing. **Timothy S. Nugent:** Resources, writing – review and editing. **Michael Durand:** Resources, writing – review and editing. **Cara Dunne:** Resources, writing – review and editing. **John O. Larkin:** Resources, writing – review and editing. **Brian Mehigan:** Resources, writing – review and editing. **Paul McCormick:** Resources, writing – review and editing. **Lorraine Brennan:** Conceptualisation, formal analysis, supervision, resources, visualisation, writing – review and editing. **Niamh Lynam-Lennon:** Conceptualisation, supervision, resources, writing – review and editing. **Jacintha O'Sullivan:** Conceptualisation, methodology, writing – original draft, writing – review and editing, supervision, resources, project administration.

## Funding source

This work is supported by an 10.13039/501100002081Irish Research Council (Grant: GOIPG/2017/983).

## Declaration of competing interest

The authors declare that they have no known competing financial interests or personal relationships that could have appeared to influence the work reported in this paper.
