# Supplementary material for: Radiation-induced Bystander Effect (RIBE) alters mitochondrial metabolism using a human rectal cancer ex vivo explant model
Source: Transl Oncol. 2020 Oct 23;14(1):100882. doi: 10.1016/j.tranon.2020.100882 (PMC7586242; doi:10.1016/j.tranon.2020.100882)
Supplement: Supplementary Table 1 — Table of metabolites discriminating NCM from irradiated normal rectal tissue compared to TCM from irradiated rectal cancer tissue. [file mmc1.docx]

**Supplementary table 1.** Table of metabolites discriminating NCM from irradiated normal rectal tissue compared to TCM from irradiated rectal cancer tissue

| Metabolite | NCM 1.8Gy | | TCM 1.8Gy | | *p*-value* |
| --- | --- | --- | --- | --- | --- |
|  | Mean | SD | Mean | SD |  |
| Acetate | 0.7827 | 0.1249 | 0.8306 | 0.2283 | 0.662 |
| Alanine | 0.7030 | 0.0635 | 0.6938 | 0.1411 | 0.708 |
| Ethanol | 1.3171 | 0.4497 | 1.8056 | 2.1846 | 0.987 |
| Isoleucine | 1.5234 | 0.1155 | 1.4831 | 0.3967 | 0.542 |
| Lactate | 3.7899 | 0.6603 | 4.0801 | 1.1393 | 0.676 |
| *Leucine* | ***2.1545*** | ***0.1728*** | ***1.9359*** | ***0.2590*** | ***0.041*** |
| Methionine | 0.3837 | 0.0305 | 0.3653 | 0.0556 | 0.328 |
| N-Acetyl-L-Alanine | 0.6235 | 0.0824 | 0.6648 | 0.1322 | 0.522 |
| Phenylalanine | 0.5868 | 0.0636 | 0.5700 | 0.0704 | 0.567 |
| Valine | 0.9712 | 0.0876 | 1.0369 | 0.1432 | 0.250 |
| *Statistical Analysis performed on log transformed variables | | | | | |
